# The danger theory in view of the injury hypothesis: 20 years later

**DOI:** 10.3389/fimmu.2012.00349

**Published:** 2012-11-26

**Authors:** Walter G. Land, Konrad Messmer

**Affiliations:** ^1^German Academy of Transplantation MedicineMunich, Germany; ^2^Former Institute for Surgical Research, University of MunichMunich, Germany

**A commentary on**

**The danger theory: 20 years later**

by Pradeu, T., and Cooper, E. L. (2012). Front. Immunol. 3:287. doi: 10.3389/fimmu.2012.00287

The “danger theory” of Polly Matzinger posed in 1994 (Matzinger, 1994) principally holds that the immune system is far less concerned with “foreign” (microorganisms, cells, and molecules) than with signals that cause dangerous damage, and, furthermore, that antigen-presenting cells respond to danger signals—most notably from cells undergoing stress and/or injury—to initiate an immune response. Of note, this theory strongly opposes the long time prevalent self-/non-self discrimination theory of immune responses that has dominated immunological thinking and acting for over 60 years.

Recently, the danger theory was emphasized in this journal by Pradeu and Cooper who assessed the topic in view of recently published experimental data (Pradeu and Cooper, 2012). When reading this article—quite admittedly with great pleasure—, we spontaneously thought to complement this thoughtfully and competently written paper by adding some notes on our “injury hypothesis” as posed in 1994/1996 (Land et al., 1994; Land and Messmer, 1996).

The injury hypothesis—which equals the danger theory—is based on stringent observations from a clinical trial in kidney transplant patients that was conducted during the late 1980s/early1990s, that is, even more than 20 years ago. Basically, the clinical data obtained from this trial showed that mitigation of postischemic allograft reperfusion injury by intraoperative injection of a single dose of the oxygen free radical scavenger “superoxide dismutase” results in a statistically significant reduction in incidence of both acute and chronic rejection events (Land et al., 1994). From these convincing clinical data, we concluded in terms of an “argumentum e contrari” that tissue injury (here: allograft injury) induces immunity (here alloimmunity).

In fact, as recently pointed out by Matzinger (Matzinger, 2012), these early clinical observations can be regarded as the true discovery of the danger model. In her article, she wrote: “*Walter Land, ex-head of experimental surgery at the Medical School, Munich, Germany was one of the first surgeons to understand the danger model. In a way, he discovered it before I published it*” (Matzinger, 2012). (Of note, in 1994 and thereafter, Walter Land was the Head of the Division of Transplantation Surgery at the Surgical University Clinic in Munich-Grosshadern/Germany).

In the same article published by us in 1994 (Land et al., 1994), we extended our concluding remarks to a working hypothesis, today known as the injury hypothesis. As illustrated and emphasized by a frame within Figure 2 of this article, a human biological immune system in its own right was proposed that is activated by non-pathogen-induced tissue injury (here: allograft reperfusion injury) and that, after activation, leads to the induction of an adaptive immune response (here: adaptive alloimmune response). In the center of this immune system, besides others, we proposed a role for antigen-presenting cells (later appreciated to be dendritic cells) activated by injury and subsequently leading to development of adaptive immunity, that is, cells operating as a bridge between injury and adaptive immunity. In other words, as from where we stand today, in 1994 we had described the existence of a human innate immune system activated by tissue injury and preceding adaptive immunity. But we missed to call the phenomenon *innate immunity*. Interestingly enough, that happened before the groups of the Nobel Laureates Jules Hoffmann (Lemaitre et al., 1996) and Bruce Beutler (Poltorak et al., 1998) published the discovery of “Toll” and “TLR4” [see also the review of Cooper (Cooper, 2010)].

During subsequent years, the injury hypothesis was extended and modified several times (Land, 2002a,b, 2003a,b, 2005). Along with these modifications, the concept of innate immunity was implemented into organ transplantation. Thus, in 2002, the possibility was discussed that allograft reperfusion injury represents a case of innate immunity that induces (“sterile”) inflammation mediated by Toll-like receptors (e.g., TLR4) interacting with heat shock proteins as their agonists (Land, 2002a,b). In the same year, we coined the term *Innate Alloimmunity* (Land, 2002a) followed by description of the term *DAMPs* in the sense of *damage-associated molecular patterns* a year later (Land, 2003b). Of note, we proposed that oxidative stress to the brain-dead donor organism as well as generation of reactive oxygen species during reperfusion of the donor organ in the recipient represent acute injurious events to the allograft that, in turn, not only lead to acute rejection but also contribute to development of chronic rejection. In particular, we suggested that activation of donor- and recipient-derived innate immune dendritic cells, via interaction of DAMPs with TLRs, leads to initiation and induction of adaptive alloimmunity and, further, activation of donor-derived innate immune vascular cells, again via interaction of DAMPs with TLRs, contributes to development of alloatherosclerosis (Land, 2002a, 2005).

In more recently published review articles, we have updated the concept of allograft injury-induced innate alloimmunity (Land, 2012a,b,c). In one of these articles, evidence is collected in support of the notion that prevention of oxidative allograft injury may operate as an efficient tool in the clinical situation to present alloantigens under subimmunogenic conditions within an intragraft non-inflammatory milieu, thereby potentially generating tolerogenic dendritic cells able to induce regulatory T cell-mediated innate allotolerance (Land, 2012c).

Finally, the whole concept of the injury hypothesis, in light of the international literature on innate immunity currently available, has been thoroughly and comprehensively discussed in a monograph that was published as two parts in 2011 (Land, 2011a,b).

We thought it would be worthwhile to provide the reader of *Frontiers in Immunology* with this information about the history of both, the danger theory and the injury hypothesis as related to organ transplantation; a note that may serve as a useful addendum to the excellent article of Pradeu and Cooper.

## References

[B1] Cooper E. L. (2010). Evolution of immune systems from self/not self to danger to artificial immune systems (AIS). Phys. Life Rev. 7, 55–78 10.1016/j.plrev.2009.12.001 20374928

[B2] Land W. Schneeberger H. Schleibner S. Illner W. D. Abendroth D. Rutili G. (1994). The beneficial effect of human recombinant superoxide dismutase on acute and chronic rejection events in recipients of cadaveric renal transplants. Transplantation 57, 211–217 8310510 10.1097/00007890-199401001-00010

[B3] Land W. Messmer K. (1996). The impact of ischemia/reperfusion injury on specific and non-specific, early and late chronic events after organ transplantation. Transplant. Rev. 10, 108–127; 236–253.

[B4] Land W. (2002a). Postischemic reperfusion injury to allografts – a case for innate immunity? Eur. Surg. Res. 34, 160–169 11867918 10.1159/000048904

[B5] Land W. (2002b). Allograft injury mediated by reactive oxygen species: from conserved proteins of Drosophila to acute and chronic rejection of human transplants. Part I: demonstration of reactive oxygen species in reperfused allografts and their role in the initiation of innate immunity. Transplant. Rev. 16, 192–204

[B6] Land W. (2003a). Allograft injury mediated by reactive oxygen species: from conserved proteins of Drosophila to acute and chronic rejection of human transplants. Part II: role of reactive oxygen species in the induction of the heat shock response as a regulator of innate immunity. Transplant. Rev. 17, 31–44

[B7] Land W. (2003b). Allograft injury mediated by reactive oxygen species: from conserved proteins of Drosophila to acute and chronic rejection of human transplants. Part III: interaction of (oxidative) stress-induced heat shock proteins with Toll-like receptor-bearing cells of innate immunity and its consequences for the development of acute and chronic allograft rejection. Transplant. Rev. 17, 67–86

[B8] Land W. G. (2005). The role of postischemic reperfusion injury and other nonantigen-dependent inflammatory pathways in transplantation. Transplantation 79, 505–514 15753838 10.1097/01.tp.0000153160.82975.86

[B9] Land W. G. (2011a). Innate Alloimmunity, Part 1: Innate Immunity and Host Defense. Ankara/Lengerich: Başkent University/Pabst Science Publishers

[B10] Land W. G. (2011b). Innate Alloimmunity, Part 2: Innate Immunity and Allograft Rejection. Ankara/Lengerich: Başkent University/Pabst Science Publishers

[B11] Land W. G. (2012a). Emerging role of innate immunity in organ transplantation: part I: evolution of innate immunity and oxidative allograft injury. Transplant. Rev. (Orlando) 26, 60–72 10.1016/j.trre.2011.05.001 22000662

[B12] Land W. G. (2012b). Emerging role of innate immunity in organ transplantation part II: potential of damage-associated molecular patterns to generate immunostimulatory dendritic cells. Transplant. Rev. (Orlando) 26, 73–87 10.1016/j.trre.2011.02.003 22074784

[B13] Land W. G. (2012c). Emerging role of innate immunity in organ transplantation part III: the quest for transplant tolerance via prevention of oxidative allograft injury and its consequences. Transplant. Rev. (Orlando) 26, 88–102 10.1016/j.trre.2011.07.001 22000661

[B14] Lemaitre B. Nicolas E. Michaut L. Reichhart J. M. Hoffmann J. A. (1996). The dorsoventral regulatory gene cassette spätzle/Toll/cactus controls the potent antifungal response in Drosophila adults. Cell 86, 973–983 10.1016/S0092-8674(00)80172-5 8808632

[B15] Matzinger P. (1994). Tolerance, danger, and the extended family. Annu. Rev. Immunol. 12, 991–1045 10.2353/ajpath.2008.070563 8011301

[B16] Matzinger P. (2012). The evolution of the danger theory. Expert Rev. Clin. Immunol. 8, 311–317 10.1586/eci.12.21 22607177 PMC4803042

[B17] Poltorak A. He X. Smirnova I. Liu M. Y. Van Huffel C. Du X. (1998). Defective LPS signaling in C3H/HeJ and C57BL/10ScCr mice: mutations in Tlr4 gene. Science 282, 2085–2088 10.1126/science.282.5396.2085 9851930

[B18] Pradeu T. Cooper E. L. (2012). The danger theory: 20 years later. Front. Immunol. 3:287 10.3389/fimmu.2012.00287 23060876 PMC3443751

